# Case Report: Improvement from no light perception after radiotherapy and surgical debulking for orbital Rosai-Dorfman disease

**DOI:** 10.3389/fopht.2025.1710976

**Published:** 2025-12-11

**Authors:** Ashlyn A. Gary, Rahul M. Dhodapkar, Sean Lim, Maria Sibug Saber, Sandy Zhang-Nunes

**Affiliations:** 1 Department of Ophthalmology, Gavin Herbert Eye Institute, University of California, Irvine, Irvine, CA, United States; 2 Department of Ophthalmology, Roski Eye Institute, Los Angeles, CA, United States; 3 Division of Rheumatology, Department of Medicine, University of Southern California Keck School of Medicine, Los Angeles, CA, United States; 4 Department of Pathology, Keck School of Medicine of the University of Southern California, Los Angeles, CA, United States; 5 Southern California Permanente Medical Group, Kaiser Permanente Los Angeles Medical Center, Los Angeles, CA, United States

**Keywords:** Rosai Dorfman disease, no light perception, compressive optic neuropathy, case report, oculoplastics

## Abstract

Rosai-Dorfman disease (RDD) is a rare proliferative histiocytic disorder with ophthalmic manifestations occurring in 11% of cases. This report details the case of a 75-year-old woman presenting with orbital RDD characterized by right-sided proptosis and progressive vision loss, culminating in no light perception (NLP) for 8 months. Imaging studies revealed tumor involvement of the right ethmoid, maxillary, and sphenoid sinuses as well as the right orbit. Biopsy confirmed extranodal RDD. The patient underwent radiotherapy, which resulted in an improvement in the visual acuity to hand motion 3 months later. Subsequent orbital decompression surgery and tumor debulking were performed to address exophthalmos and worsening exposure keratopathy. Postoperatively, visual acuity improved to counting fingers at 6 in. in the first week and further to 20/800 seven months after surgery. Vision-threatening compressive optic neuropathy is a severe complication of orbital RDD; however, this case demonstrates its potential reversibility with a multidisciplinary therapeutic approach.

## 1 Introduction

Rosai-Dorfman disease (RDD) is a rare, idiopathic disorder characterized by histiocyte proliferation, with an estimated annual incidence of 100 new cases in the United States (1, 2). Typically, in benign and often self-limiting conditions, RDD predominantly affects children and young adults, manifesting most commonly as bilateral, painless cervical lymphadenopathy (1). The precise etiology of RDD remains largely undefined. Hypotheses include a dysregulatory immune response following exposure to infectious agents such as bacteria (e.g., *Klebsiella*) and viruses (e.g., Parvovirus B19, Cytomegalovirus, Epstein–Barr virus, and Human Herpesvirus 6) (3). Conversely, some theories challenge the purely reactive nature of RDD, proposing that certain instances may represent a neoplastic-like process. Mutations commonly associated with various cancers, including BRAF V600E, KRAS, and MAP2K1 L115V, have identified a subset of RDD cases (4–8). However, definitive causal links between infectious and neoplastic processes in RDD have yet to be established.

While RDD exhibits a predilection for the head and neck lymph nodes, 43% of patients have extranodal involvement in areas such as the skin, paranasal sinuses, bones, and eyes (9). Approximately 11% of RDD cases present with ocular signs and symptoms, but isolated ophthalmic disease without lymphadenopathy is exceedingly rare (10, 11). Ophthalmic manifestations most typically involve the orbit, presenting as exophthalmos, which can lead to decreased visual acuity, reduced ocular motility, diplopia, and dry eye disease (9, 10). Intraocular manifestations including scleritis, uveitis, choroidal mass, serous retinal detachment, and lacrimal duct obstruction have also been documented (11–13). In this case report, we describe a patient with orbital RDD who had 8 months of confirmed no light perception (NLP) vision, which improved following external radiation therapy, orbital decompression, and tumor debulking. To the best of our knowledge, the occurrence of visual recovery in patients with long-standing NLP secondary to compressive optic neuropathy in RDD has not been reported previously. Patients with NLP vision secondary to mass-induced stretch optic neuropathy may retain greater potential for visual recovery after tumor debulking than previously thought. This report highlights the potential for visual recovery following NLP and contributes to the ongoing discourse on the clinical and surgical management of orbital RDD.

## 2 Case description

**Figure 1:**
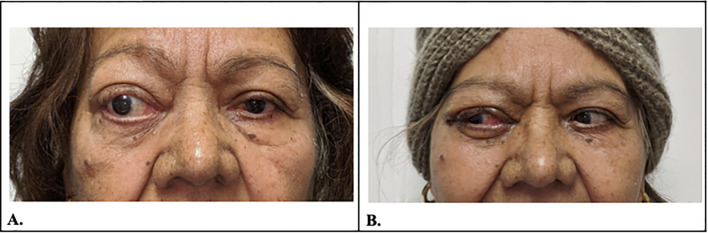
**(A)** Photo demonstrating significant proptosis in the right eye. **(B)** 1-week post-operative photo demonstrating resolved right eye proptosis.

**Figure 2:**
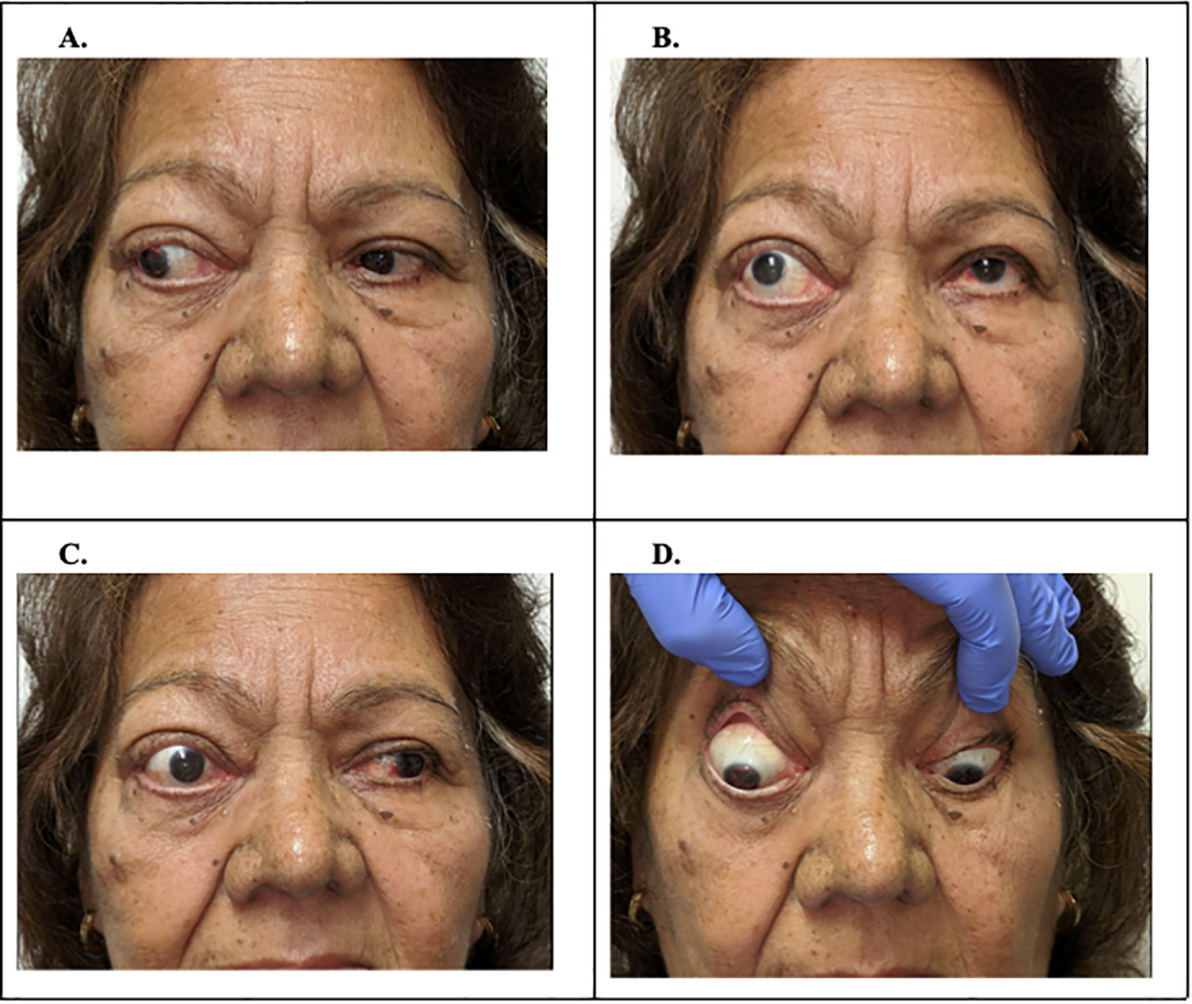
Patient’s ocular motility in four gaze positions (left **(A)**, up **(B)**, right **(C)**, down **(D)**, with adduction deficit demonstrated in right eye **(C)**.

A 75-year-old Hispanic woman with a medical history of hypertension, congestive heart failure, and severe primary open-angle glaucoma in both eyes was referred to our center for surgical consultation of a single contiguous tumor invading the right ethmoid, maxillary, and sphenoid sinuses and right orbit. On examination, she exhibited significant exophthalmos in her right eye (Marco 98 base, 25 right eye, and 19 left eye) (**Figure 1A**). She also demonstrated a -2 to -3 adduction deficit in her right eye (**Figure 2C**). Visual acuity was no light perception (NLP) right eye and 20/25 left eye. The intraocular pressure was 19 mmHg in the right eye and 21 mmHg in the left eye. Examination of the right eye and eyelid demonstrated severe upper lid retraction, blepharitis, and 2+ inferior superficial punctate keratitis (SPK) in the cornea. The anterior chamber was normal, and the iris revealed anisocoria with a nonreactive 6 mm pupil, demonstrating an afferent defect. A 3+ nuclear sclerotic cataract is observed in the lens. The left eye, orbit, and adnexa were within normal limits. Right eye posterior segment examination revealed a cup-to-disc (C/D) ratio of 0.95 with optic nerve cupping and a minimal superior rim. In the left eye, the C/D ratio was 0.85 with optic nerve cupping.

**Figure 3:**
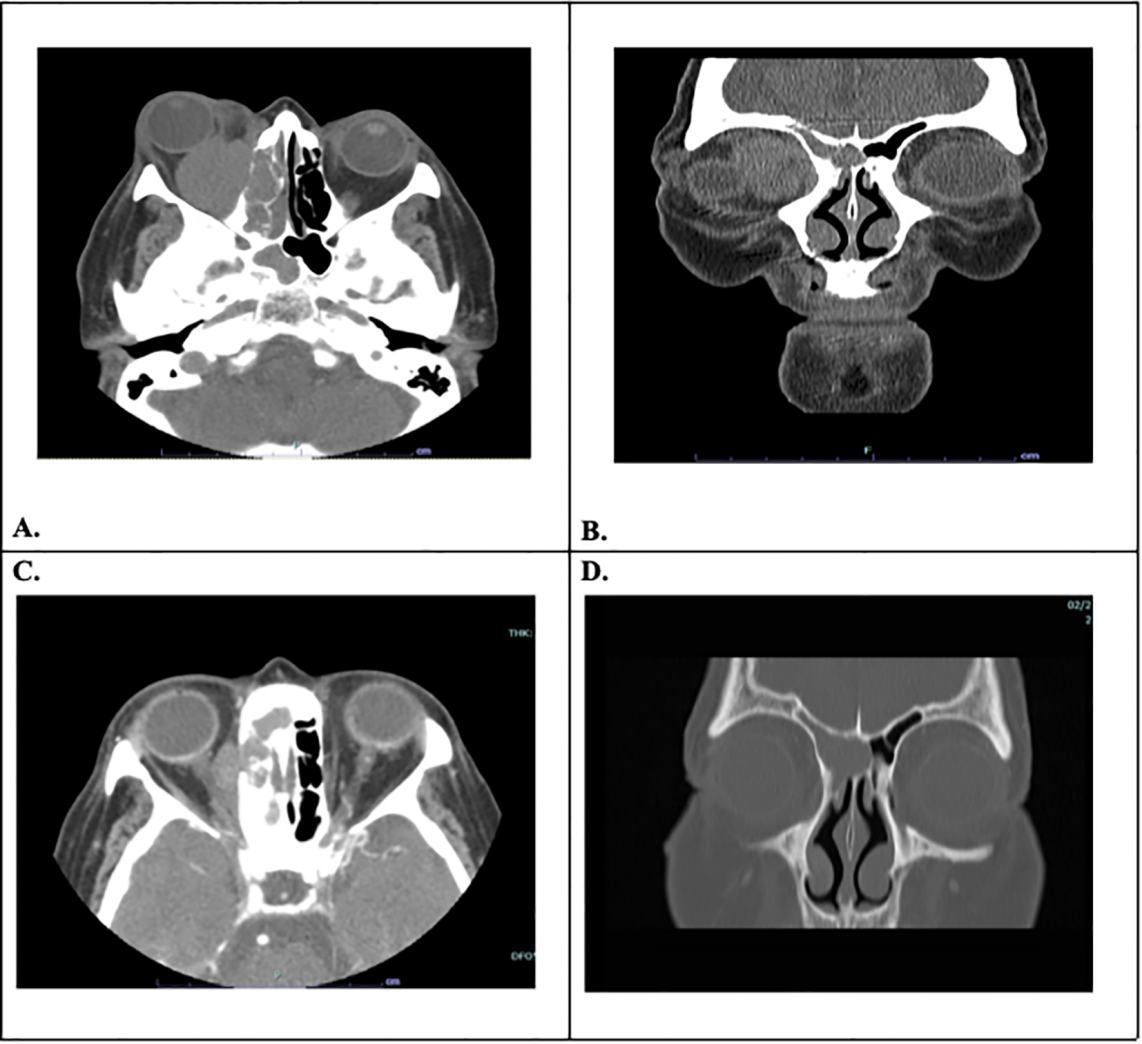
**(A)** Axial high-resolution CT showing orbital infiltration with right optic nerve compression. **(B)** Coronal high-resolution CT showing orbital infiltration. **(C)** Axial high-resolution 1-month post-operative CT. **(D)** Coronal high-resolution 1-month post-operative CT.

The patient’s symptoms started three years prior to her first presentation, initially with binocular diplopia, and then progressed to frankly visible proptosis and vision loss. According to the patient, her symptoms were both aesthetically disfiguring and disruptive to her daily functioning and quality of life. The patient was being followed up by an ophthalmologist for glaucoma and eventually underwent maxillofacial CT, which identified an orbital nasal sinus mass (**Figure 3****).** The mass was subsequently biopsied and confirmed to be RDD. A PET scan also demonstrated a hypermetabolic irregularly shaped infiltrating mass arising in the right nasal cavity extending into the right orbit with an ethmoid air cell appearance highly consistent with neoplastic pathology. There were also right-sided hypermetabolic cervical lymph nodes measuring 16 mm × 18 mm. Upon review of symptoms, the patient denied lymphadenopathy and sinus-related issues, including nasal obstruction, epistaxis, congestion, and pain. She also reported no constitutional symptoms such as fever, night sweats, or weight loss.

## 3 Diagnostic assessment

**Figure 4:**
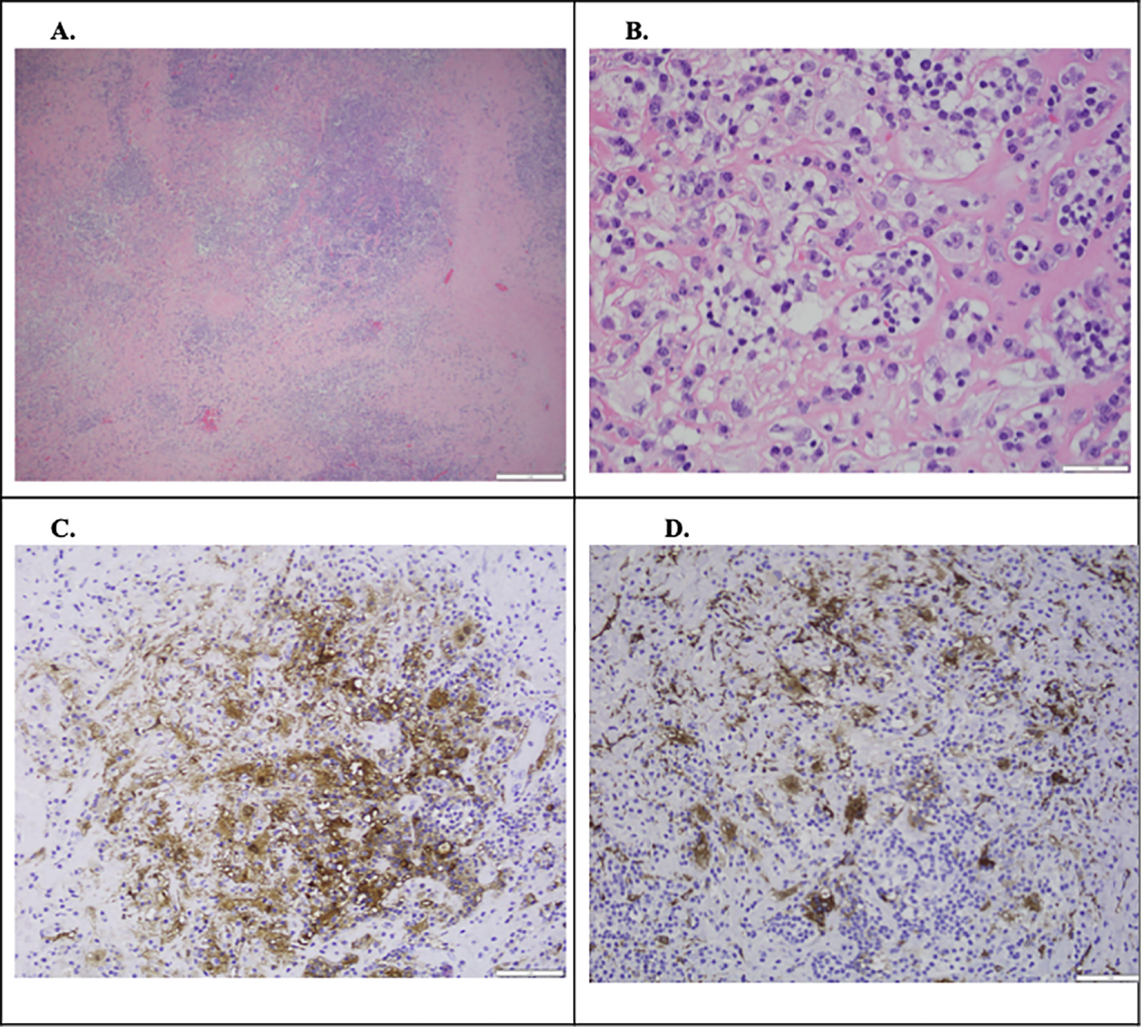
Pathology confirmed the presence of extranodal Rosai-Dorfman disease (RDD). **(A)** H&E, ×4: overview showing dense fibrosis with lymphoid cell infiltration and aggregates of foamy histiocytes (paler areas). **(B)** H&E, ×40: high-power view demonstrating emperipolesis. **(C)** S100 immunostain, ×20: highlights histiocytes. **(D)** CD163 immunostain, ×20: confirms histiocytic lineage.

The patient had been confirmed to have NLP vision by two ophthalmologists eight months before the initial visit to our institution. The patient completed 40 Gy of external radiation over four weeks one month prior to presentation, with minimal self-reported improvement when she started perceiving light in her right eye. This improvement in visual acuity was corroborated by two seasoned ophthalmologists who confirmed light perception with direction and bare-hand motion vision in the right eye. Owing to the severity of proptosis and exposure keratopathy, the surgical team and patient decided to pursue palliative surgery to debulk the tumors. On 28 January 2020, the patient underwent a right orbitotomy with a bone flap via a lateral approach for lesion removal, a right orbitotomy without a bone flap via a conjunctival approach for the medial superior and inferior quadrants, right ethmoidectomy, and right canthoplasty. The tumor was situated in the inferonasal quadrant of the orbit, eroded through the medial wall, and extended into the nasal cavity. Pathology confirmed the presence of extranodal Rosai-Dorfman disease (RDD), characterized by dense fibrosis with infiltration by small lymphocytes, numerous polyclonal plasma cells, and multifocal aggregates of S100+ foamy histiocytes, exhibiting focal emperipolesis (**Figure 4**). One week postoperatively, the patient’s right eye vision improved to counting fingers at 6 in., and her left eye vision was 20/30. The intraocular pressure was 10 mmHg in the right eye and 21 mmHg in the left eye. Exophthalmometry measured 98 bases, with 18 mm in the right eye and 17.5 mm in the left eye, indicating a significant reduction in proptosis (**Figure 1B**). Seven months postoperatively, the patient’s right eye vision had improved to 20/800. Following surgery, the patient was very grateful for regaining significantly more vision in her right eye and appreciated the improvement in her proptosis. Four years postoperatively, the patient’s right eye vision declined to hand motion, with recurrent Rosai-Dorfman disease in the sinuses, as confirmed by the latest CT scan. The pupillary reaction improved, but mild APD persisted. Additionally, her glaucoma worsened, particularly in the right eye (right eye > left eye), which was attributed to mixed-mechanism optic neuropathy secondary to a compressive mass lesion and open-angle glaucoma.

## 4 Discussion

Orbital RDD can lead to significant visual complications. Our patient presented with proptosis, diminished visual acuity, restricted eye movement, and exposure keratitis, all of which were attributable to histiocytic infiltration of the orbit and optic nerve compression, respectively. These clinical manifestations are consistent with previously described presentations described in the literature. For instance, a multicenter study involving eight patients with histopathologically confirmed ophthalmic RDD reported that the orbit was the most frequently involved site (90.9% of eyes), with proptosis observed in 90% of cases (14). Our patient exhibited an atypical clinical course, characterized by the absence of subjective lymphadenopathy with initial symptoms of diplopia, vision loss, and proptosis, followed by an unexpected improvement in long-standing no-light perception (NLP) vision. Although the patient likely had pre-existing glaucoma, we propose that the condition worsened due to mixed-mechanism optic neuropathy secondary to a compressive mass lesion. To the best of our knowledge, one study reported an association of uveitic glaucoma and RDD (15). Although improvements in visual acuity following RDD treatment have been documented (14, 16), concurrent cases of glaucoma and vision loss to NLP have not been described, and there are no documented cases of reversing NLP in the context of RDD.

Cases of RDD with compressive optic neuropathy have been previously documented (12, 17). In the literature, vision-threatening compressive optic neuropathy has served as an indication of chemotherapy in ophthalmic RDD (12, 17). A promising treatment approach involves the combination of chemotherapeutic agents targeting both B cells (e.g., rituximab) and T cells (e.g., methotrexate and cyclosporine) (12). In the aforementioned multicenter comprehensive study, two patients presented with secondary optic neuropathy. Despite the initial treatment with oral steroids and surgical debulking, the disease remained unstable. Subsequent referral to oncology and the introduction of chemotherapy resulted in disease stabilization, with no progression reported for over two years (14). In contrast, our patient did not receive steroids or chemotherapy but demonstrated improvement in visual symptoms with external radiation and surgical debulking. The management of orbital RDD patients is not standardized and frequently necessitates an individualized, multidisciplinary approach.

Recovery from NLP is exceedingly rare, and NLP secondary to compressive optic neuropathy lasting >48 h is generally considered a poor prognostic indicator (18). Nonetheless, only a few case reports on visual recovery have been published. For example, a case series involving three patients with severe visual impairment due to Graves’ optic neuropathy demonstrated significant improvement following orbital decompression surgery (ranging from NLP to 20/400, counting fingers to 20/20, and NLP to 20/60) (19). The patients in these studies exhibited NLP or counting fingers (CF) for up to three months; in contrast, our patient presented with NLP for eight months. Similarly, Stark et al. reported a 9-year-old girl who, after experiencing NLP for several months due to optic nerve compression from a craniopharyngioma, improved to 20/40 vision following decompression surgery (20). Bampoe et al. also reported a patient who improved from NLP after resection of a large skull base meningioma (21). Research utilizing a feline model of optic nerve compression has suggested that nerve conduction can be disrupted through various mechanisms including hypoxia, depolarization, cytoskeleton disruption, demyelination, and axotomy (22). Based on their model, Cottee et al. concluded that remyelination or regeneration of cytoskeletal components (microtubules) was the most probable reason for functional recovery. Although rare, recovery from NLP can be explained biologically by the potential for some degree of axonal regeneration after decompression. Radiation and surgery can initiate regeneration by alleviating pressure on the optic nerve, reducing inflammation, and restoring blood flow. Our case study indicates that axonal death may not occur up to 8 months after total nerve function loss, and that decompression and tumor debulking may still be effective in patients with prolonged NLP, thereby highlighting the potential for partial recovery in cases where complete visual loss is often considered a contraindication for surgical intervention.

## 5 Conclusion

In conclusion, Rosai-Dorfman disease should be considered in patients presenting with proptosis and orbital masses. Treating orbital RDD is challenging because of its rarity and the absence of standardized approaches, often necessitating individualized, multidisciplinary treatment strategies involving specialties, such as radiation oncology, otorhinolaryngology, and oculoplastics. Our case report demonstrates that radiotherapy, decompression, and tumor debulking may still be effective in RDD patients with prolonged NLP.

## Data Availability

The original contributions presented in the study are included in the article/supplementary material. Further inquiries can be directed to the corresponding author.

## References

[1] Rosai J Dorfman RF . Sinus histiocytosis with massive lymphadenopathy. A newly recognized benign clinicopathological entity. Arch Pathol. (1969) 87:63–70., PMID: 5782438

[2] Abla O Jacobsen E Picarsic J Krenova Z Jaffe R Emile JF . Consensus recommendations for the diagnosis and clinical management of Rosai-Dorfman-Destombes disease. Blood. (2018) 131:2877–90., PMID: 29720485 10.1182/blood-2018-03-839753 PMC6024636

[3] McClellan SF Ainbinder DJ . Orbital Rosai-Dorfman disease: a literature review. Orbit. (2013) 32:341–6. doi: 10.3109/01676830.2013.814689, PMID: 23895540

[4] Badalian-Very G Vergilio JA Degar BA MacConaill LE Brandner B Calicchio ML . Recurrent BRAF mutations in Langerhans cell histiocytosis. Blood. (2010) 116:1919–23., PMID: 20519626 10.1182/blood-2010-04-279083 PMC3173987

[5] Hervier B Haroche J Arnaud L Charlotte F Donadieu J Néel A . Association of both Langerhans cell histiocytosis and Erdheim-Chester disease linked to the BRAFV600E mutation. Blood. (2014) 124:1119–26., PMID: 24894769 10.1182/blood-2013-12-543793

[6] Choi MB Salomão DR Smith WM Pulido JS Garrity JA . Ophthalmic findings of rosai-dorfman disease. Am J Ophthalmol. (2018) 188:164–72. doi: 10.1016/j.ajo.2018.01.037, PMID: 29428455

[7] Shanmugam V Margolskee E Kluk M Giorgadze T Orazi A . Rosai-dorfman disease harboring an activating KRAS K117N missense mutation. Head Neck Pathol. (2016) 10:394–9. doi: 10.1007/s12105-016-0709-6, PMID: 26922062 PMC4972763

[8] Matter MS Bihl M Juskevicius D Tzankov A . Is Rosai-Dorfman disease a reactve process? Detection of a MAP2K1 L115V mutation in a case of Rosai-Dorfman disease. Virchows Arch. (2017) 471:545–7. doi: 10.1007/s00428-017-2173-4, PMID: 28597077

[9] Foucar E Rosai J Dorfman R . Sinus histiocytosis with massive lymphadenopathy (Rosai-Dorfman disease): review of the entity. Semin Diagn Pathol. (1990) 7:19–73., PMID: 2180012

[10] Vemuganti GK Naik MN Honavar SG . Rosai dorfman disease of the orbit. J Hematol Oncol. (2008) 1:7. doi: 10.1186/1756-8722-1-7, PMID: 18588698 PMC2474646

[11] Foucar E Rosai J Dorfman RF . The ophthalmologic manifestations of sinus histiocytosis with massive lymphadenopathy. Am J Ophthalmol. (1979) 87:354–67. doi: 10.1016/0002-9394(79)90077-1, PMID: 434097

[12] Mohadjer Y Holds JB Rootman J Wilson MW Gigantelli JW Custer PL . The spectrum of orbital Rosai-Dorfman disease. Ophthalmic Plast Reconstr Surg. (2006) 22:163–8. doi: 10.1097/01.iop.0000217563.00975.a3, PMID: 16714922

[13] Pivetti-Pezzi P Torce C Colabelli-Gisoldi RA Vitale A Baccari A Pacchiarotti A . Relapsing bilateral uveitis and papilledema in sinus histiocytosis with massive lymphadenopathy (Rosai-Dorfman disease). Eur J Ophthalmol. (1995) 5:59–62. doi: 10.1177/112067219500500110, PMID: 7795403

[14] Alzahem TA Cruz AA Maktabi AMY Chahud F Alkatan H . Ophthalmic Rosai-Dorfman disease: a multi-centre comprehensive study. BMC Ophthalmol. (2021) 21:404. doi: 10.1186/s12886-021-02173-1, PMID: 34814862 PMC8609855

[15] MacLaren RE Hundal KS Trittibach P Bloom PA . Uveitic glaucoma and Rosai-Dorfman disease (sinus histiocytosis). Ocul Immunol Inflamm. (2006) 14:305–7. doi: 10.1080/09273940600878829, PMID: 17056465

[16] Su X Zhang L . Orbital Rosai-Dorfman disease: a case report and literature review. J Int Med Res. (2019) 47:5891–5. doi: 10.1177/0300060519878086, PMID: 31612761 PMC6862901

[17] Goldberg S Mahadevia P Lipton M Rosenbaum PS . Sinus histiocytosis with massive lymphadenopathy involving the orbit: reversal of compressive optic neuropathy after chemotherapy. J Neuroophthalmol. (1998) 18:270–5. doi: 10.1097/00041327-199812000-00010, PMID: 9858010

[18] Waybright EA Selhorst JB Young HF Harbison JW . Tumors compressing the optic nerve: diagnosis and surgical results. Va Med. (1983) 110:230–4., PMID: 6868781

[19] Devoto MH Golnik K Bernardini FP Alencar VM . Improvement from no light perception after orbital decompression for graves’ optic neuropathy. Ophthalmology. (2014) 121:431–2.e1. doi: 10.1016/j.ophtha.2013.09.031, PMID: 24268859

[20] Stark KL Kaufman B Lee BC Primack J Tychsen L . Visual recovery after a year of craniopharyngioma-related amaurosis: report of a nine-year-old child and a review of pathophysiologic mechanisms. J aapos. (1999) 3:366–71., PMID: 10613582 10.1016/s1091-8531(99)70047-9

[21] Bampoe J Ranalli P Bernstein M . Postoperative reversal of complete (monocular) blindness in skull base meningioma: case report. Can J Neurol Sci. (2003) 30:72–4. doi: 10.1017/S0317167100002481, PMID: 12619789

[22] Cottee LJ Daniel C Loh WS Harrison BM Burke W . Remyelination and recovery of conduction in cat optic nerve after demyelination by pressure. Exp Neurol. (2003) 184:865–77. doi: 10.1016/S0014-4886(03)00310-8, PMID: 14769379

